# Three-Dimensional-Printed Biomimetic Structural Ceramics with Excellent Tribological Properties

**DOI:** 10.3390/ma18061376

**Published:** 2025-03-20

**Authors:** Zhaozhi Wang, Yajie Liu, Biao Jiang, Zhiheng Xin, Zhibin Jiao

**Affiliations:** School of Mechanical Engineering, Shenyang University of Technology, Shenyang 110870, China; zhaozhi_wang@sut.edu.cn (Z.W.); ddliuyajie@163.com (Y.L.); 13364513802@163.com (B.J.); 16688375411@163.com (Z.X.)

**Keywords:** 3D printing, bioinspired ceramics, solid lubricants, wear-resistant

## Abstract

Inspired by the ventral scale structure of the oriental sand boa, this study successfully fabricated multiscale bioinspired alumina (Al_2_O_3_) ceramics by combining the excellent mechanical properties, high-temperature resistance, and high hardness of ceramic composites with direct ink writing (DIW) 3D printing technology and femtosecond laser processing. A MoS_2_ thin film was then deposited on the ceramic surface via radio frequency magnetron sputtering (PVD) to systematically investigate the impact of bioinspired structures on the tribological properties of ceramic composites under both dry and lubricated conditions. Experimental results demonstrated that bioinspired structures at different scales exhibited significant friction-reducing and wear-resistant characteristics compared to blank structures. Specifically, under room-temperature conditions, the friction coefficients of bioinspired ceramic composites with solid lubricants and oil lubrication were 0.3 and 0.148, respectively, indicating excellent tribological performance. These findings confirm the synergistic lubrication effect between bioinspired structures, two-dimensional solid lubricants, and lubricating oil, which significantly enhanced the friction-reducing and wear-resistant properties of ceramic components. Therefore, the synergistic design of multiscale bioinspired structures and solid lubricants provides an innovative strategy for the advanced application of ceramic components.

## 1. Introduction

During mechanical operation, friction and wear phenomena inevitably occur due to the interaction forces between components, leading to significant energy losses. These losses have become one of the primary contributors to energy consumption. In recent years, with increasingly stringent national requirements for energy conservation, emission reduction, and environmental protection, reducing frictional losses has emerged as a critical issue in the industrial sector. Alumina (Al_2_O_3_) ceramics exhibit excellent high-temperature resistance, corrosion resistance, high hardness, superior wear resistance, and creep resistance, showing broad application potential in fields such as aerospace, precision electronics, biomedical devices, and military equipment [1,2,3,4]. However, the inherent brittleness and poor machinability of ceramic materials severely limit their application in the manufacturing of complex structures [5]. Traditional alumina ceramic fabrication techniques, such as gel casting, die pressing, and injection molding, face numerous technical challenges when producing ceramics with complex geometries [6]. Additionally, alumina ceramics exhibit high wear rates and friction coefficients under dry friction conditions, further restricting their practical application in mechanical moving components [7].

In recent years, the rapid development of 3D printing technology has provided innovative solutions for the fabrication of ceramic components. This technology overcomes the geometric limitations of traditional manufacturing processes, enabling the freeform fabrication of complex structures. Additionally, 3D printing offers rapid, mold-free manufacturing capabilities, avoiding the potential damage caused by secondary processing in conventional methods and facilitating the digital design and fabrication of all components [8]. Various 3D printing technologies have been successfully applied to the rapid prototyping of ceramic materials, including stereolithography (SLA) [9], digital light processing (DLP) [10], direct ink writing (DIW) [11], and fused deposition modeling (FDM) [12]. Among these, the DIW technique, with its unique room-temperature extrusion deposition process, significantly reduces the effects of thermal and residual stresses [13], demonstrating notable advantages in versatility and applicability [14]. These characteristics make DIW a cost-effective and efficient method, particularly suitable for fabricating ceramics with complex structures [15]. Therefore, compared to traditional ceramic manufacturing methods, the integration of DIW 3D printing technology with ceramic materials offers an efficient and convenient approach for designing and fabricating complex ceramic structures, providing significant engineering application value [16].

Through millions of years of natural selection and evolution, many organisms have developed wear-resistant surface structures adapted to extreme environments, such as snake scales, pangolin scales, sand lizard scales, and intertidal mollusk shells [17]. The exceptional tribological properties exhibited by these biological surfaces have significantly inspired research within the academic community. Biomimetic tribology aims to develop materials with bioinspired structures and functions by thoroughly investigating the friction-reducing, wear-resistant, and efficient lubrication mechanisms of typical organisms. To date, the role of biomimetic design in improving the tribological properties of materials has been extensively validated through both theoretical research and experimental studies [18,19]. For example, Li et al. [20] inspired by the toe structure of tree frogs, optimized the parameters of bioinspired hexagonal structures on AISI 4140 steel surfaces using response surface methodology. The results demonstrated that the optimized bioinspired textured samples reduced the average friction coefficient by 20.82% and the wear track depth by 65.65% compared to non-textured samples, fully confirming the significant effectiveness of bioinspired structures in reducing friction and wear. Chen [21] designed biomimetic petal structures and tree frog toe-inspired ceramic structures using 3D printing technology based on natural biological features. Experimental results showed that the bioinspired structures not only exhibited lower friction coefficients but also achieved more uniform stress distribution, effectively reducing edge stress concentration. Additionally, Li et al. [22] fabricated biomimetic sharkskin structures on Al_2_O_3_/TiC ceramic surfaces and deposited WS_2_ coatings. Compared to nonstructured ceramics, the bioinspired structured ceramics achieved an 84% reduction in the friction coefficient. With the increasing demands for friction and wear performance in industrial applications, the integration of bioinspired structures with ceramic 3D printing technology holds significant engineering value for reducing energy consumption, minimizing friction losses, extending the service life of ceramic components, and enhancing their reliability.

To further explore the excellent tribological performance of bioinspired structural ceramics in various applications, combining bioinspired structures with solid lubricant coatings has proven to be an effective approach [23]. Solid lubricants are materials capable of providing lubrication under nearly dry conditions [24]. Composite lubrication structures primarily utilize surface bioinspired textures as carriers for solid lubricants, thereby offering continuous lubrication replenishment to friction pairs. Additionally, the surface textures themselves exhibit friction-reducing and wear-resistant properties, enabling synergistic lubrication effects with solid lubricants [25]. Previous studies have shown that the synergistic interaction between bioinspired textures and solid lubricants offers significant advantages in reducing friction coefficients and wear rates [26,27,28]. These bioinspired structures demonstrate excellent performance in lubricant storage, altering the contact mode of debris at friction interfaces, and reducing the contact area, thereby significantly enhancing the wear resistance of composite materials and improving the efficiency and durability of mechanical components [29]. Multiscale surface topography plays a crucial role in determining the tribological behavior of additively manufactured ceramic composites, with wettability effects influencing frictional performance, as observed in polymer-based additive manufacturing studies [30]. However, current research has primarily focused on the preparation of solid lubricant coatings on metal or alloy surfaces, while studies on ceramic surfaces remain insufficient and require further investigation.

Based on this, the present study proposes an innovative strategy for fabricating Al_2_O_3_/ZrO_2_/MoS_2_ composites by integrating the 3D printing of bioinspired structures with solid lubricant coating technology. Inspired by the ventral scale structure of the oriental sand boa and based on the theory of biomimetic tribology, we used the macroscopic and microscopic morphology of the abdominal scales of the oriental sand oyster as a biomimetic blueprint to establish a coupled biomimetic model that integrates both macroscopic and microscopic structures. Various Al_2_O_3_ ceramic bioinspired structures were designed and fabricated using direct ink writing (DIW) technology and femtosecond laser processing. This study systematically investigates the synergistic lubrication effects and mechanical properties of the bioinspired structures and solid lubricants, spanning macro to micro scales. Furthermore, the microstructure, phase composition, wear mechanisms, and lubrication mechanisms of the composites are thoroughly analyzed and discussed, providing theoretical foundations and practical guidance for subsequent optimization designs.

## 2. Materials and Methods

### 2.1. Bionic Wear-Resistant Prototype and Structural Design

**Figure 1:**
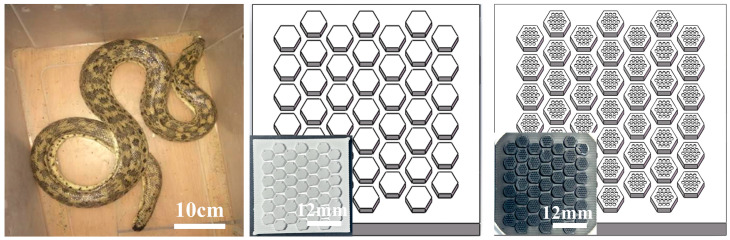
Bionic prototype and 3D-printed bionic ceramic model and image after sintering [36].

Figure 1 illustrates images of bioinspired, bionic models, as well as 3D-printed bioinspired ceramic structures after sintering. The ventral scales of snakes, which directly interact with the external environment during movement, exhibit macrostructures and surface microtextures resulting from long-term environmental adaptation, endowing the scales with exceptional tribological properties. The snake scales display a regular hexagonal structure, with dense nanoscale pits distributed across the microscopic surface. This unique microstructure facilitates the formation of a thin lubricating film between contact surfaces, effectively reducing direct surface contact. The presence of pits not only reduces the contact area and minimizes atomic adhesion between sand particles and the surface, thereby alleviating abrasive wear, but also disperses stress and inhibits crack propagation, further mitigating surface damage. Inspired by this, a bioinspired model combining a hexagonal macrostructure and micro-pits was developed based on the morphological features of the oriental sand boa’s ventral scales. Orthogonal experimental results indicated that a regular hexagon with a side length of 2 mm yielded a relatively low friction coefficient. Consequently, the side length of the hexagonal macrostructure was set to 2 mm, with the spacing and depth of the bioinspired units set to 1 mm.

Furthermore, texture density significantly influences the tribological performance of the samples. Under dry friction conditions, texture density directly affects the contact area at the interface, as well as the capture and storage efficiency of abrasive particles [31], while under lubricated conditions, it influences the distribution of contact pressure [32]. Wu [33] investigated the effect of pit density on the tribological behavior of titanium alloys, revealing that the spacing between adjacent pits significantly impacts the friction coefficient and wear rate of textured surfaces. Specifically, reducing pit spacing enhances the friction and wear performance of surfaces filled with solid lubricants. Schneider [34] demonstrated that laser-textured pits with a density of 10% could minimize friction force. Meanwhile, Ezhilmaran [35] found that laser-textured piston rings with a pit density of 16% significantly reduced the friction coefficient, and concluded that the friction coefficient increases significantly with higher pit density. To eliminate the effects of friction anisotropy, the micro-pits in this study were designed in a ring-shaped, evenly spaced arrangement, with a diameter of approximately 200 μm, a depth of about 50 μm, and a spacing of 100 μm.

### 2.2. Preparation of Ceramic Slurry for 3D Printing

In this experiment, spherical α-Al_2_O_3_ with an average particle size of 500 nm (99% purity, Anhui Yishi Tong Materials Technology Co., Ltd., Hefei, China) and 8Y-ZrO_2_ (99% purity, Shanghai Zhongye New Materials Co., Ltd., Shanghai, China) were selected as the ceramic powder raw materials. Sodium alginate (Ron Reagents, Kunshan, China) was used as a thickener, polyvinylpyrrolidone (Hefei BASF Biotechnology Co., Ltd., Hefei, China) as a binder, sodium hexametaphosphate (Tianjin Hengxing Chemical Reagent Manufacturing Co., Ltd., Tianjin, China) as a curing agent, and ammonium polyacrylate (Dow Chemical Company, Midland, MI, USA) as a dispersant to modify the ceramic powders. Deionized water prepared in the laboratory was used as the solvent.

First, a ceramic slurry premix was prepared. Polyvinylpyrrolidone (2 wt% of the ceramic powder mass), sodium hexametaphosphate (2 wt%), sodium alginate (0.3 wt%), ammonium polyacrylate (3 wt%), and deionized water (22 wt%) were sequentially added to a mixing tank and blended at 600 rpm for 20 min using a planetary centrifugal mixer (ZD-T600C, Shenzhen Zhidi Technology Co., Ltd., Shenzhen, China). Subsequently, alumina (Al_2_O_3_) and zirconia (ZrO_2_) ceramic powders were mixed at a molar ratio of 2:1 and ground in a planetary ball mill at 400 rpm for 2 h to ensure uniform mixing. Prior to mixing, the Al_2_O_3_ and ZrO_2_ powders were dried in a vacuum oven (BPZ-6033, Shanghai Yiheng Technology Co., Ltd., Shanghai, China) at 100 °C for 24 h to remove internal moisture. After preparing the premix, the ceramic powder was added to the mixing tank in small batches and mixed at 800 rpm using the planetary centrifugal mixer. The final ceramic slurry, with a solid content of 77.5%, was obtained after mixing. The slurry was then transferred to a syringe using a spatula and degassed under vacuum. Before filling, the viscosity of the slurry was measured at room temperature (25 °C) using a viscometer (NDJ-8S, Shanghai Lichen Bangxi Instrument Technology Co., Ltd., Shanghai, China) at 150 rpm to ensure it met the printing requirements of the direct ink writing (DIW) 3D printer. The prepared ceramic slurry exhibited shear-thinning behavior, characteristic of non-Newtonian fluids, to meet the demands of the printing process.

### 2.3. Preparation of 3D-Printed Bionic Structural Ceramics

**Figure 2:**
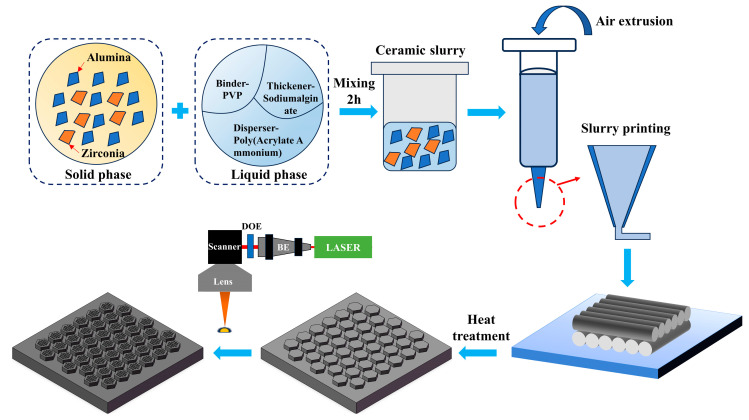
Preparation process of biomimetic ceramic composite materials.

**Figure 3:**
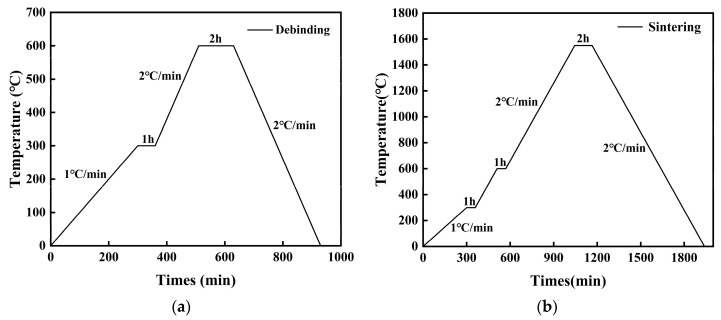
Three-dimensional-printed ceramic raw billet heat treatment process curve. (**a**) Debinding curve; (**b**) sintering curve.

This study utilized SolidWorks software (Version 30.0.0.5020, Dassault Systèmes, Vélizy-Villacoublay, France) to design bioinspired structural samples with dimensions of 30 × 30 × 4 mm^3^. The model data were saved in stereolithography (STL) format and then imported into a direct ink writing (DIW) 3D printer (Bio-architect SR, Hangzhou Genefly Biotech Co., Ltd., Hangzhou, China) for slicing and subsequent printing. Figure 2 illustrates the preparation process of the biomimetic ceramic composite materials. Through multiple experimental optimizations, the nozzle size and printing parameters were determined: a 0.41 mm nozzle, with a layer height of 0.32 mm, printing speed of 10 mm/s, and air pressure of 0.25 MPa. After printing, the green bodies were removed from the printing platform and air-dried naturally for 24 h. The dried samples then underwent sequential debinding and sintering processes. Debinding was performed in a debinding pre-sintering furnace (FX2CT-60/11, Hefei Fischer Thermal Engineering Equipment Co., Ltd., Hefei, China), while sintering was conducted in a high-temperature sintering furnace (FMJ-12/18, Hefei Fischer Thermal Engineering Equipment Co., Ltd., Hefei, China). Since the densification of pressure-less-sintered Al_2_O_3_ ceramics primarily relies on high-temperature grain boundary diffusion to bond ceramic powder particles, a temperature control protocol was developed, as shown in Figure 3. This protocol ensures that the ceramic materials achieve desirable microstructures and mechanical properties during sintering.

This study employed femtosecond laser processing (Anyang Laser Co., Ltd., Wuhan, China) technology to fabricate micro bioinspired structures on the surface of macro bioinspired units. The femtosecond laser processing parameters were set as follows: central wavelength of 1064 nm, frequency of 0.5 kHz, pulse number of 3, pulse width of 100 ns, processing cycles of 3, processing speed of 500 mm/s, with air as the processing environment. Figure 1 shows the model of the 3D-printed bioinspired structure and the top-view optical image after heat treatment. As shown in the figure, the material surface exhibits a tightly arranged structure without cracks, and both the dimensional design and debinding–sintering processes are well controlled, with no macroscopic defects observed. These characteristics contribute to enhancing the mechanical properties and wear resistance of the ceramics. However, a deviation of approximately 400 μm exists between the 3D-printed bioinspired ceramic samples and the designed dimensions. This deviation primarily arises from the following factors: (1) variations in drying time between layers during the layer-by-layer printing process, (2) inhomogeneity in the slurry curing process, and (3) inconsistent shrinkage rates in different directions during the debinding and sintering processes. Table 1 lists the bulk density and basic performance parameters of the bioinspired structural samples, providing critical references for further process optimization.

### 2.4. Preparation of Solid Lubricant Coatings

In this experiment, a molybdenum disulfide (MoS_2_) coating was deposited onto the surface of 3D-printed bioinspired ceramic composites using physical vapor deposition (PVD) technology (Shenzhen Pulang Innovation Technology Co., Ltd., Shenzhen, China), specifically employing a three-target magnetron sputtering deposition system. In the experimental setup, the MoS_2_ target was positioned at the bottom of the vacuum chamber, while the substrate was placed at the top. The fixture size was set to φ50 mm × 3 mm, the initial vacuum level reached 7 × 10^−4^ Pa, and the target-to-substrate distance was adjusted to 25.8 mm. To ensure uniform film deposition, the bioinspired ceramic samples were rotated at 15 rpm, resulting in a MoS_2_ film with a thickness of approximately 1.5 µm. The specific process parameters are listed in Table 2. During deposition, the MoS_2_ target was sputtered using a radio frequency (RF) power supply. Prior to deposition, the polished bioinspired ceramic composites were ultrasonically cleaned in anhydrous ethanol for 20 min and then air-dried to ensure that surface cleanliness met the deposition requirements.

### 2.5. Tribological Performance Testing

**Figure 4:**
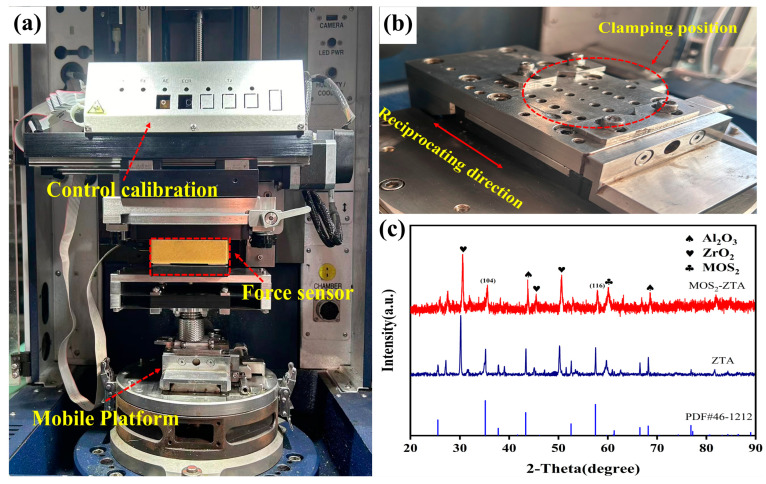
(**a**) Linear reciprocating friction tester; (**b**) clamping device; 3D-printed ceramics; (**c**) XRD diffractograms before and after MoS_2_ coating.

This study utilized a Bruker multi-functional friction and wear tester (UMT-5 TriboLab, Bruker Physik-AG, Karlsruhe, Germany) to conduct reciprocating dry friction tests on bioinspired ceramic structural samples, with the experimental setup shown in Figure 4a. A corundum ceramic plate, prepared by static pressure sintering, was chosen as the friction pair. The plate had a purity of 99%, dimensions of 12 mm × 10 mm × 5 mm, a surface roughness (Ra) of 0.1 μm, and a Vickers hardness (MHV-30, Suzhou Jinsong Metrology Instrument Co., Ltd., Suzhou, China) of 1700 Hv. The experiments were conducted at room temperature (25 °C) under a load of 20 N, with a frequency of 3 Hz, amplitude of 15 mm, and a test duration of 1800 s per run. Each test was repeated three times to ensure reliable data. To systematically evaluate the tribological performance of the bioinspired structures, a single-factor experimental design was adopted, comparing the following three groups of samples: (1) macro bioinspired samples (T, T-O, and T-S); (2) macro/micro bioinspired samples (TP, TP-O, and TP-S); and (3) blank samples (UT, UT-O, and UT-S). By comparing the friction and wear performance of samples with different structures, the influence of bioinspired designs on the tribological behavior of the materials was analyzed. X-ray diffraction (XRD) (AL-Y3500, Dandong Aolong Radiative Instrument Group Co., Ltd., Dandong, China) was used to examine the crystal structure and phase composition of the ceramic samples. The testing conditions were as follows: Cu Kα radiation, a scanning speed of 5°/min, a step size of 0.02°, a tube voltage of 30 kV, a tube current of 30 mA, and a scanning range from 20° to 90°. XRD analysis provided insights into the crystal structure and any phase composition changes in the samples.

To explore the morphological features of the friction surfaces and the evolution mechanisms of the contact interfaces, scanning electron microscopy (SEM) (SEM3100, CIQTEK Co., Ltd., Hefei, China) was employed to characterize the surfaces before and after friction testing. SEM analysis revealed the microscopic features of the worn surfaces, offering critical insights into the wear mechanisms. Additionally, to quantitatively assess the wear performance of the samples, a high-precision electronic balance was used to weigh the samples before and after the wear tests, and the wear volume was calculated based on the mass loss.

## 3. Results

### 3.1. Microscopic Morphology and Phase Composition Analysis

The surface of the 3D-printed bioinspired structural samples was characterized using scanning electron microscopy (SEM) and X-ray diffraction (XRD) to examine their micro-morphology and compositional features. Figure 4c presents the surface morphology and microstructure of the Al_2_O_3_/ZrO_2_/MoS_2_ ceramic composite. It shows spherical, nano-sized alumina and zirconia particles, uniformly dispersed and agglomerated, indicating strong dispersion and bonding between the two ceramic materials at the microscopic level.

**Figure 5:**
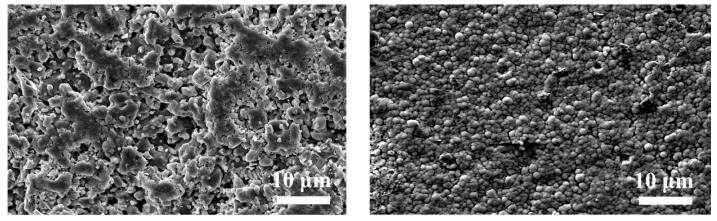
Initial SEM surface morphology (**left**); surface morphology containing MoS_2_ (**right**).

Figure 5 displays the micro-morphology of the MoS_2_ solid lubricant deposited on the surface of the bioinspired samples. As shown, MoS_2_ is uniformly deposited without any missed areas, demonstrating good coverage and consistency throughout the deposition process. Figure 4c presents the X-ray diffraction (XRD) pattern of the samples. According to the standard card (PDF46-1212), the sharp and high-intensity diffraction peaks of Al_2_O_3_ and ZrO_2_ indicate that both materials exhibit excellent crystallinity after heat treatment. Notably, the characteristic peak of ZrO_2_ near 30° indicates a phase transformation from the monoclinic phase (m-ZrO_2_) to the tetragonal phase (t-ZrO_2_), which significantly influences the material’s mechanical properties.

### 3.2. Tribological Performance Analysis

**Figure 6:**
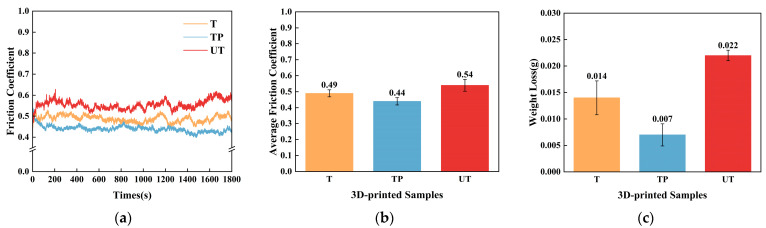
Friction performance profiles of 3D-printed bionic structural ceramics under dry friction conditions. (**a**) Friction coefficient curve; (**b**) average friction coefficient; (**c**) weight loss.

**Figure 7:**
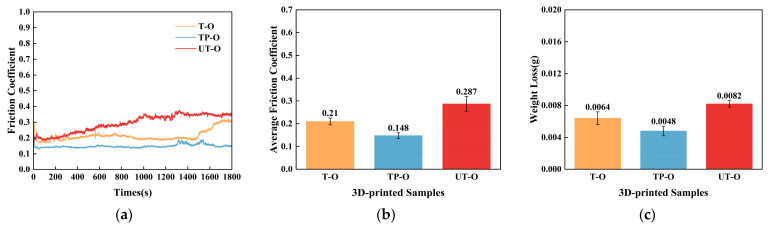

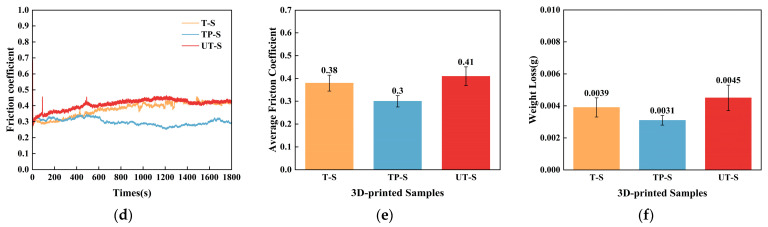
Friction performance profiles of 3D-printed bionic structural ceramics under lubricated friction conditions. (**a**,**d**) Friction coefficient curve; (**b**,**e**) average friction coefficient; (**c**,**f**) weight loss.

To investigate the tribological performance of 3D-printed bioinspired ceramic composites with different scales, this study conducted a qualitative analysis by measuring the friction coefficient. Figure 5 and Figure 6 illustrate the variation in friction coefficients under different friction media for three structural categories: nonstructured surfaces (UT, UT-O, and UT-S), macro bioinspired structures (T, T-O, and T-S), and macro/micro bioinspired structures (TP, TP-O, and TP-S), tested at a friction frequency of 3 Hz. As shown in Figure 6a, both the T and UT samples exhibited a significant initial increase in friction coefficient. Specifically, the friction coefficient of the UT sample rapidly increased from an initial value of 0.2 to 0.6, before gradually decreasing and stabilizing. In contrast, the TP sample exhibited a higher friction coefficient initially, which may be attributed to the formation of a molten layer at the edges of the pits due to the high temperatures involved in femtosecond laser processing. From 0 to 120 s, the friction coefficient of the TP sample decreased, likely due to the formation of micro-lubrication pools in the friction contact area created by the pit structures. These pools temporarily store lubricants (e.g., air) and reduce the friction coefficient while effectively dispersing contact stress. Additionally, compared to the T-O sample, the TP-O sample showed more stable fluctuations in the friction coefficient. Notably, the T-O sample experienced a sharp increase in the friction coefficient around 1500 s due to insufficient lubrication (see Figure 7a), whereas the TP-O sample demonstrated more consistent tribological performance. The experimental results suggest that the synergistic effect of bioinspired macro/microstructures significantly enhances the tribological properties, especially under lubricated conditions. As shown in Figure 7b, both T and TP specimens with biomimetic structures exhibit superior wear resistance compared to the unstructured specimen (UT), with significantly lower wear rates.

Further analysis of Figure 7a reveals that the friction coefficients of the UT-O and T-O specimens exhibited a monotonically increasing trend over time, while the TP-S specimen demonstrated a distinctive “N”-shaped friction coefficient profile. Specifically, the TP-S specimen shows significant fluctuations at 500 s, followed by a continuous increase in the friction coefficient after 1200 s, which may be due to lubrication failure from film wear. Notably, around 1700 s, a secondary decrease in the friction coefficient is observed, likely caused by the continuous release of solid lubricant stored in the biomimetic dimple structures under pressure, establishing an effective secondary lubrication mechanism. Quantitative analysis indicates that the average friction coefficients of the TP-S and TP-O specimens are reduced by 44.4% and 72.6%, respectively, compared to the UT specimen, conclusively demonstrating the significant enhancement of tribological performance through bioinspired structural design.

The experimental data further reveal that the biomimetic-structured T and TP ceramic specimens exhibit significantly lower average friction coefficients and wear rates than conventional UT specimens under both dry and lubricated conditions. This conclusively demonstrates the efficacy of biomimetic design in enhancing the wear resistance of ceramic materials. Notably, although oil-based lubricants exhibit lower friction coefficients than the solid lubricant MoS_2_ at ambient temperature, their application is substantially limited: they fail to maintain stable lubrication under starved conditions and are prone to volatilization or depletion in vacuum or low-pressure environments, leading to lubrication failure. The results highlight a remarkable synergistic effect between the biomimetic macro/microstructures and lubricating media. This synergy not only effectively reduces friction coefficients but also significantly enhances the wear resistance of ceramic composites. These findings provide a novel technical approach for expanding the engineering applications of ceramic materials in complex working conditions.

### 3.3. Wear Surface Morphology Analysis

**Figure 8:**
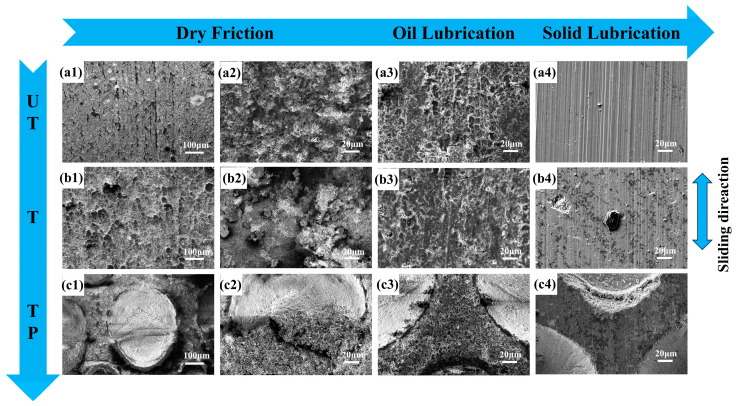
SEM morphology of wear surfaces of 3D-printed bionic structural ceramic materials: Dry friction condition (**a1**,**a2**) UT specimen, (**b1**,**b2**) T specimen, (**c1**,**c2**) TP specimen; Oil lubrication condition (**a3**) UT specimen, (**b3**) T specimen, (**c3**) TP specimen; Solid lubrication condition: (**a4**) UT specimen, (**b4**) T specimen, (**c4**) TP specimen.

To investigate the wear mechanisms and lubrication behaviors of ceramic composites with multiscale biomimetic structures, the worn surfaces were systematically characterized using scanning electron microscopy (SEM) following friction tests. Comparative analysis revealed that the wear tracks of the T (Figure 8(b1,b2)) and TP specimens (Figure 8(c1,c2)) exhibited significantly less damage than those of the UT specimens (Figure 8(a1,a2)). The worn surface of the UT specimens displayed typical localized damage, such as particle pull-out, irregular oxide layers, and distinct delamination, indicating substantial surface damage during friction. In contrast, the wear morphology of the T specimens was primarily characterized by intergranular fractures and particle pull-out, arising from increased intergranular cracking due to cyclic stress induced by normal force.

Notably, while UT specimens showed discontinuous plastic deformation layers, the T specimens demonstrated effective stress concentration relief due to their macroscopic biomimetic structures, significantly enhancing their friction reduction and wear resistance. These findings highlight the critical role of biomimetic structural design in improving the anti-wear performance of ceramic materials under dry friction conditions. Under oil lubrication (Figure 8(a3,b3,c3)), the worn surfaces of oil-lubricated specimens (T-O) showed brighter coloration and uneven crack distribution compared to dry-friction specimens (T), indicating that microcrack propagation was the primary wear mechanism. High-magnification observation of partially worn dimple regions (Figure 8(c2)) revealed that some wear debris was captured by the dimples during friction, effectively reducing abrasive wear [37]. Furthermore, as wear debris reached the edges of the biomimetic dimple structures and came into contact with the friction pair, torque was generated, causing debris rotation and creating a “bearing-like” effect that transformed sliding friction into rolling friction. This unique friction conversion mechanism likely explains the lower friction coefficient fluctuations observed in the TP specimens compared to the T specimens.

Biomimetic structures significantly modify the mechanical properties of contact interfaces; however, the edges of the biomimetic dimples can induce localized stress concentration during sliding friction, particularly at the edges of the microscopic biomimetic units where stress concentration is more pronounced. Under cyclic stress, the accumulation of adhesive fatigue leads to material spalling at the textured edges, resulting in micro-cutting wear. Importantly, the presence of local hexagonal biomimetic structures provides effective stress support, preventing the spread of stress concentration across the entire specimen surface, thereby reducing more severe stress damage. Under oil lubrication conditions (Figure 8(a3,b3,c3)), the scouring effect of the lubricating oil resulted in minimal abrasive particle accumulation near the wear tracks. The surface of the single-component MoS_2_ coating exhibited a characteristic loose dendritic morphology with distinct columnar features in cross-section [38] (Figure 5). Under solid lubrication conditions, all specimens demonstrated significant plowing effects on their worn surfaces, with UT-S specimens showing the most pronounced plowing effect and localized lubricant film spalling observed, as shown in Figure 8(b4). As sliding time increased, the gradual depletion of solid lubricant caused a continuous rise in the friction coefficient [39]. During reciprocating friction, the failure of the lubricating layer and rupture of the lubricant film were the primary reasons for the increased friction coefficient in the TP-S specimens at 1200 s. Additionally, wear debris acting as a third body in the friction process adhered to the worn surface under compression, leading to surface plowing (Figure 8(c4)), which was another significant factor contributing to fluctuations in the friction coefficient [40,41].

## 4. Discussion

The ternary synergistic effect of macro/micro biomimetic structures combined with the MoS_2_ solid lubricant coating significantly reduces the friction coefficient and enhances the wear resistance of ceramic components. To elucidate the lubrication mechanisms and wear behavior of these biomimetic ceramic composites, Figure 8 presents a schematic diagram that illustrates these mechanisms. This schematic systematically demonstrates the multiple functional roles of biomimetic structures during the friction process:

(1) Macroscopic hexagonal biomimetic units effectively disperse contact stress, reducing local stress concentration.
(2) Microscopic dimple structures serve as lubricant reservoirs, releasing lubricant during friction and establishing long-lasting secondary lubrication effects.
(3) MoS_2_ solid lubricant coating forms a stable lubricating film at the friction interface, significantly lowering the interfacial friction coefficient.
(4) Stress concentration mitigation: The local support provided by the biomimetic structures effectively reduces stress at their edges, preventing crack initiation and propagation.
(5) Debris-trapping and rolling effects: The dimple structures capture wear debris and induce rolling effects, reducing abrasive wear and further enhancing wear resistance.

**Figure 9:**
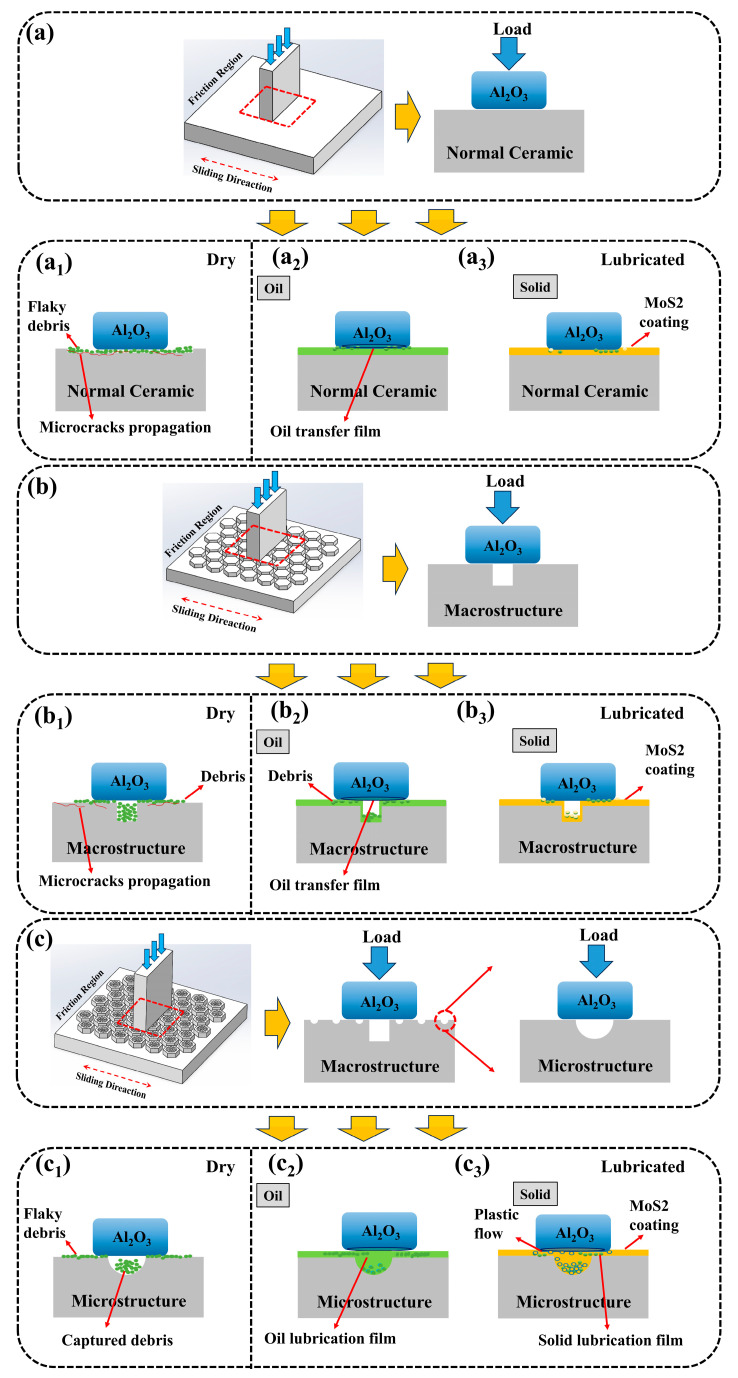
Schematic diagram of lubrication and wear mechanism of 3D-printed bionic structure specimens. (**a**) UT specimen, (**a1**) Dry friction conditions, (**a2**) Oil lubrication conditions, (**a3**) Solid lubrication conditions; (**b**) T specimen, (**b1**) Dry friction conditions, (**b2**) Oil lubrication conditions, (**b3**) Solid lubrication conditions; (**c**) TP specimen, (**c1**) Dry friction conditions, (**c2**) Oil lubrication conditions, (**c3**) Solid lubrication conditions.

Under dry friction conditions, the blank UT structure is more prone to generating and accumulating abrasive debris when sliding against harder Al_2_O_3_ ceramic plates. Interlayer cracks form readily on the contact surface under cyclic stress, and as these cracks propagate and intersect, material delaminates into flaky debris, causing severe abrasive and fatigue wear (Figure 9(a2)). The presence of macroscopic biomimetic units reduces the contact area and alleviates stress concentration on the friction surface, inhibiting crack propagation caused by cyclic stress. Additionally, the intercellular groove structure reduces debris accumulation at the friction interface, mitigating surface plowing and decreasing the likelihood of three-body wear (Figure 9(b2)). Through the synergistic effect of macro/micro biomimetic units, not only is the contact area reduced, but the debris-trapping capacity is also enhanced (Figure 9(c2)), significantly improving the material’s wear resistance.

According to relevant research [42], the friction coefficient (*μ*) between two smooth surfaces under elastic loading conditions can be expressed as

(1) $$\mu =A\frac{\tau }{F^{1/3}}({\frac{3}{4E^{\prime }})}^{2/3}$$

where *A* is a constant determined by the contact geometry, *τ* represents the shear stress at the contact interface, *F* is the applied normal load, and *E*′ is the elastic modulus of the substrate material. Under oil lubrication conditions, the biologically inspired grooves between the macroscopic biomimetic units not only provide effective channels for lubricant flow but also act as lubricant reservoirs. As friction progresses, the lubricant forms a continuous film at the contact interface, significantly reducing interfacial shear stress and effectively suppressing crack propagation caused by stress concentration. The lubricating film minimizes direct material contact, dissipates heat, and removes wear debris, thus optimizing tribological performance. Additionally, the microscopic biomimetic units provide extra storage space for the lubricant, enabling gradual release during material compression and wear, further reducing the wear rate and friction coefficient.

The unique structural features of the microscopic biomimetic units enhance the adhesion of the MoS_2_ solid lubricant to the material surface [43]. During reciprocating friction, a uniform solid lubricating film with low shear strength forms on the substrate surface, transforming the contact interface into friction between a hard substrate and a soft lubricating layer. This reduces shear stress at the contact surface, allowing the hard substrate to bear the external load while the lubricating layer minimizes direct contact between hard substrates, resulting in a significantly reduced friction coefficient, wear rate, and abrasive wear of the ceramic specimens, demonstrating excellent friction reduction and wear resistance [44]. However, as friction continues, the lubricating film gradually delaminates, leading to the formation of flaky fragments on the friction contact surface. When the lubricating film reaches its load-bearing limit and ruptures, direct contact occurs between the friction pairs, causing a sharp increase in the friction coefficient, consistent with the experimental results shown in Figure 7d. Notably, the solid lubricant embedded in the microscopic biomimetic units provides secondary lubrication after the lubricating film ruptures, extending the service life of the MoS_2_ coating and enhancing the material’s overall tribological performance.

## 5. Conclusions

This study successfully designed and fabricated ceramic composites with biomimetic hexagonal structures using 3D printing technology, combined with physical vapor deposition (PVD) to deposit MoS_2_ solid lubricant on the surface, systematically investigating the tribological properties of biomimetic structures under both dry and lubricated conditions. The research reveals the synergistic mechanism between macro/micro biomimetic units and lubricating media, providing an innovative strategy for optimizing the wear resistance of ceramic materials. Experimental results demonstrate that the biomimetic structures significantly reduce the friction coefficient and wear rate, while the introduction of solid lubricant further enhances the material’s lubrication performance. This study provides a theoretical foundation and technical support for the engineering applications of ceramic materials under complex working conditions and draws the following conclusions:

(1) The introduction of biomimetic structures significantly optimizes the tribological properties of the matrix material. Compared to blank specimens, biomimetic-structured specimens demonstrate a reduction in the average friction coefficient by 44.4% and 72.6% under dry and lubricated conditions, respectively, exhibiting excellent friction reduction and wear resistance. The synergistic effect between macroscopic hexagonal biomimetic structures and microscopic dimples significantly reduces the contact area and stress concentration, effectively inhibiting crack propagation, thereby substantially enhancing the wear resistance of ceramic composites.
(2) A notable synergistic effect exists between biomimetic structures and lubricating media. Under oil lubrication conditions, biomimetic structures provide ideal storage and circulation spaces for lubricant, promoting the formation of a stable lubricating film. The incorporation of the MoS_2_ solid lubricant coating further enhances the material’s lubrication performance, particularly achieving a long-lasting secondary lubrication effect in the microscopic biomimetic units.
(3) The material’s wear mechanism undergoes a significant transformation. The synergistic design of biomimetic structures and solid lubricant provides an innovative and efficient solution for the high-end applications of ceramic materials, not only significantly improving wear resistance and service life but also demonstrating substantial engineering application value.

## Figures and Tables

**Table 1:** Mechanical properties of ceramic materials.

Bulk Density (g·cm^−3^)	Flexural Strength (MPa)	Fracture Toughness (Mpa/m^2^)	Vickers Hardness (HV)	Line Shrinkage (%)		
4.70	126.657	2.702	172.10	X	Y	Z
				6.13	5.96	6.94

**Table 2:** Preparation parameters of MoS_2_ composite thin film.

Samples	Ar Flow (sccm)	RF Power (W)	Deposition Time (min)
UT-S	45	150	120
T-S	45	150	120
TP-S	45	150	120

## Data Availability

The original contributions presented in this study are included in the article. Further inquiries can be directed to the corresponding author.
